# Expression levels of the DNA repair enzyme HAP1 do not correlate with the radiosensitivities of human or HAP1-transfected rat cell lines

**DOI:** 10.1038/sj.bjc.6690447

**Published:** 1999-06

**Authors:** C J Herring, B Deans, R H Elder, J A Rafferty, J MacKinnon, G Barzilay, I D Hickson, J H Hendry, GP Margison

**Affiliations:** CRC Section of Genome Damage and Repair Paterson Institute for Cancer Research, Christie Hospital (NHS) Trust, Manchester, M20 4BX, UK; Genome Integrity Group, ICRF Institute of Molecular Medicine, University of Oxford, John Radcliffe Hospital, Oxford, OX3 9DS, UK

**Keywords:** ionizing radiation, apurinic sites, oxidative DNA damage, APEX, APE, Ref-1

## Abstract

Apurinic/apyrimidinic (AP) sites in DNA are potentially lethal and mutagenic. They can arise spontaneously or following DNA damage from reactive oxygen species or alkylating agents, and they constitute a significant product of DNA damage following cellular exposure to ionizing radiation. The major AP endonuclease responsible for initiating the repair of these and other DNA lesions in human cells is HAP1, which also possesses a redox function. We have determined the cellular levels of this enzyme in 11 human tumour and fibroblast cell lines in relation to clonogenic survival following ionizing radiation. Cellular HAP1 levels and surviving fraction at 2 Gy (SF2) varied five- and tenfold respectively. However, no correlation was found between these two parameters following exposure to γ-irradiation at low (1.1 cGy per min) or high (108 cGy per min) dose rates. To examine this further, wild-type and mutant versions of HAP1 were overexpressed, using an inducible HAP1 cDNA expression vector system, in the rat C6 glioma cell line which has low endogenous AP endonuclease activity. Induction of wild-type HAP1 expression caused a > fivefold increase in the capacity of cellular extracts to cleave an oligonucleotide substrate containing a single abasic site, but increased expression did not confer increased resistance to γ-irradiation at high- or low-dose rates, or to the methylating agent methyl methanesulphonate (MMS). Expression in C6 cell lines of mutant forms of HAP1 deleted for either the redox activator or DNA repair functions displayed no apparent titrational or dominant negative effects. These studies suggest that the levels of endogenous AP endonuclease activities in the various cell lines examined are not limiting for efficient repair in cells following exposure to ionizing radiation or MMS. This contrasts with the correlation we have found between HAP1 levels and radiosensitivity in cervix carcinomas (Herring et al (1998) *Br J Cancer* **78**: 1128–1133), indicating that HAP1 levels in this case assume a critical survival role and hence that established cell lines might not be a suitable model for such studies. © 1999 Cancer Research Campaign


*British Journal of Cancer* (1999) **80**(7), 940–945
© 1999 Cancer Research Campaign
Article no. bjoc.1998.0447

### Expression levels of the DNA repair enzyme HAP1 do not correlate with the radiosensitivities of human or HAP1-transfected rat cell lines

**CJ Herring[1,*], B Deans[1,†], RH Elder[1], JA Rafferty[1], J MacKinnon[1], G Barzilay[2], ID Hickson[2], JH Hendry[1] and GP Margison[1]**

[1]CRC Section of Genome Damage and Repair, Paterson Institute for Cancer Research, Christie Hospital (NHS) Trust, Manchester M20 4BX, UK; [2]Genome Integrity Group, ICRF Institute of Molecular Medicine, University of Oxford, John Radcliffe Hospital, Oxford OX3 9DS, UK

**Summary** Apurinic/apyrimidinic (AP) sites in DNA are potentially lethal and mutagenic. They can arise spontaneously or following DNA damage from reactive oxygen species or alkylating agents, and they constitute a significant product of DNA damage following cellular exposure to ionizing radiation. The major AP endonuclease responsible for initiating the repair of these and other DNA lesions in human cells is HAP1, which also possesses a redox function. We have determined the cellular levels of this enzyme in 11 human tumour and fibroblast cell lines in relation to clonogenic survival following ionizing radiation. Cellular HAP1 levels and surviving fraction at 2 Gy (SF2) varied five- and tenfold respectively. However, no correlation was found between these two parameters following exposure to γ-irradiation at low (1.1 cGy per min) or high (108 cGy per min) dose rates. To examine this further, wild-type and mutant versions of HAP1 were overexpressed, using an inducible HAP1 cDNA expression vector system, in the rat C6 glioma cell line which has low endogenous AP endonuclease activity. Induction of wild-type HAP1 expression caused a > fivefold increase in the capacity of cellular extracts to cleave an oligonucleotide substrate containing a single abasic site, but increased expression did not confer increased resistance to γ-irradiation at high- or low-dose rates, or to the methylating agent methyl methanesulphonate (MMS). Expression in C6 cell lines of mutant forms of HAP1 deleted for either the redox activator or DNA repair functions displayed no apparent titrational or dominant negative effects. These studies suggest that the levels of endogenous AP endonuclease activities in the various cell lines examined are not limiting for efficient repair in cells following exposure to ionizing radiation or MMS. This contrasts with the correlation we have found between HAP1 levels and radiosensitivity in cervix carcinomas (Herring et al (1998) *Br J Cancer* **78**: 1128–1133), indicating that HAP1 levels in this case assume a critical survival role and hence that established cell lines might not be a suitable model for such studies.

**Keywords:** ionizing radiation; apurinic sites; oxidative DNA damage; APEX; APE; Ref-1

The base-excision repair pathway has evolved to repair a diverse range of base modifications and base losses as well as damage to the sugar-phosphate backbone of DNA. This damage may arise through spontaneous base hydrolysis (Lindahl and Anderson, 1972), endogenous oxidative metabolism (Lindahl, 1990), or exposure to genotoxic agents, including agents that exert their effects via the formation of reactive oxygen species, such as peroxides and ionizing radiation (reviewed by Demple and Harrison, 1994).

The major cytotoxic lesion following exposure of cells to ionizing radiation is considered to be the DNA double-strand break (dsb) associated with other lesions in damage complexes termed local-multiply (or regional)-damaged sites (Ward et al, 1994). However, apurinic/apyrimidinic (AP) sites are major lesions resulting from the direct effects of radiation on DNA bases or following glycosylase-mediated excision of specific modified bases. The action of AP endonucleases on these AP sites produces single-strand breaks that are likely to contribute to the formation of dsb if they are in close proximity to other lesions. Disruption of the sugar-phosphate backbone of DNA also occurs causing strand breaks with atypical termini (Teoule, 1987). These include 3′ phosphate and 3′-phosphoglycolate termini which cannot be used as primers for DNA synthesis by *Escherichia coli* DNA polymerase I or T4 DNA polymerase (Henner et al, 1983), since a 3′ hydroxyl terminus is required. Consequently, these 3′ terminal modified deoxyribose moieties constitute blocks to DNA repair synthesis.

If unrepaired, both AP sites and 3′ blocked termini can be toxic (Demple et al, 1986), and AP sites can also be mutagenic (Loeb and Preston, 1986). The major cellular enzymes responsible for initiating the repair of these sites are class II AP endonucleases (reviewed by Doetsch and Cunningham, 1990; Demple and Harrison, 1994; Barzilay and Hickson, 1995). AP endonucleases incise the phosphodiester backbone 5′ to the abasic site to generate 3′ phosphate and 5′ deoxyribose phosphate termini. Additionally, the 3′-diesterase activity processes blocked 3′ termini to produce 3′ hydroxyl termini (Demple and Harrison, 1994; Winters et al, 1994). The 5′ deoxyribose phosphate group is excised by a deoxyribose phosphodiesterase, giving 5′ phosphate, and the single nucleotide gap thus generated can be filled by a DNA polymerase and repair completed by a DNA ligase.

In human cells the major AP endonuclease is HAP1 (Robson and Hickson, 1991); also termed APEX, APE and Ref-1 (Demple

*Received 18 August 1998*
*Revised 3 November 1998*
*Accepted 11 November 1998*

*Correspondence to:* GP Margison

*Present address: Department of Pathology, University of Cambridge, Tennis Court Road, Cambridge CB2 1QP, UK
†Present address: Radiation and Genome Stability Unit, MRC Harwell, Didcot, Oxon OX11 0RD, UK

et al, 1991; Seki et al, 1991; Xanthoudakis and Curran, 1992). HAP1 possesses AP endonuclease, 3′ phosphatase and 3′ phosphodiesterase DNA repair activities and RNAase H activity (reviewed in Barzilay et al, 1996; Rothwell et al, 1997). In addition, HAP1 has been shown to function as a 'redox' modifier, facilitating the DNA binding of *fos* and *jun*, and other transcription factors, through reductive activation (Xanthoudakis et al, 1992). This activity may implicate HAP1 in a gene regulatory role, coordinating cellular responses to oxidative and/or hypoxic stress. HAP1 also is a potent activator of p53 by both redox-dependent and -independent means, and it can stimulate p53 transactivation in vivo (Jayaraman et al, 1997). The DNA repair and redox transcriptional regulator functions of the enzyme are dependent upon distinct active sites (Walker et al, 1993; Xanthoudakis et al, 1994). The conserved amino acids Glu-96, Asp-283, His-309 and Asn-212 are essential for efficient DNA repair activity (Barzilay et al, 1995*a*, 1995*b*; Rothwell and Hickson, 1996), while Cys-65 is crucial for redox activity (Walker et al, 1993).

It is, therefore, not unreasonable to suggest that the activity of AP endonucleases may contribute to cell survival following exposure to ionizing radiation. However, the amounts of enzyme required to deal with endogenously generated damage (see above) are likely to be small in comparision to the amounts required to deal with the substantial levels of damage from therapeutic doses of ionizing radiation, and therefore normal expression levels might be expected to be rate-limiting in the repair of AP sites in irradiated cells.

Several studies have shown that cellular depletion of HAP1 protein via expression of antisense HAP RNA sensitizes cells to killing by a wide range of cytotoxic agents, including methyl methanesulphonate (MMS) and peroxides (Ono et al, 1994; Walker et al, 1994), the redox cycling drug menadione, the radiomimetic agent bleomycin (Barzilay et al, 1996), and X-rays (Chen and Olkowski, 1994). Also, a correlation between HAP1 levels and the sensitivity of a series of glioma cell lines to MMS or hydrogen peroxide was reported, using the dose for 50% survival as the sensitivity parameter (Ono et al, 1995). In addition, there was a trend towards a correlation between HAP1 levels and the sensitivity of the same series of cell lines to X-rays. Although the correlation ($r = 0.71$) was found to be not statistically significant ($P = 0.11$), a subsequent re-analysis of the same published data, using the linear-quadratic model and the alpha parameter as a measure of radiosensitivity, produced a significant ($P = 0.02$) and higher degree of correlation (P Lambin, personal communication). Furthermore, a correlation has recently been demonstrated for the levels of HAP1 immunostaining of cervical carcinoma sections and the in vitro radiosensitivity of primary cells cultured from such tumours ($r = 0.60$, $P = 0.002$; Herring et al, 1998). The possibility that HAP1 expression might be used to contribute to the prediction of the clinical response of normal and tumour tissues to radiotherapy encouraged investigation of the possible contribution of HAP1 to the varying radiosensitivity observed among a further series of human cell lines. In parallel, radiation responses were also determined following overexpression of wild-type and mutated HAP1 in a mammalian cell line with low endogenous AP endonuclease activity.

### MATERIALS AND METHODS

### Normal cell lines

Du145 (human prostate carcinoma), WiDr (human colon adenocarcinoma), A2780 and HOC8 (human ovarian carcinoma), Ms751 and ME180 (human cervical carcinoma), NB1-g and NB1-clone F (human neuroblastoma), cells were cultured in basal modified Eagle's medium (MEM) supplemented with 10% fetal calf serum (FCS). The cell lines 350s, 351s and vag12 (normal human fibroblast), were cultured in MEM with 15% FCS; and C6 (rat glioma) cells in Dulbecco's modified Eagle's medium (DMEM; Gibco) with 10% FCS. Cells were maintained in exponential growth at 37°C in an atmosphere of 5% carbon dioxide.

### Cells containing inducible wild-type or mutant HAP1

The HAP1 cDNA (or the mutated derivatives HAP1-C6SA and HAP1-D283A; Walker et al, 1993; Barzilay et al, 1995*a*) was amplified using the polymerase chain reaction with 5′ and 3′ primers which contain *Not*1 recognition sites. In addition, the 5′ primer contained the sequence coding for the c-*myc* epitope recognized by the 9E10 monoclonal antibody. Following digestion of the product with *Not*1, the DNA was ligated into a *Not*1-digested pOPRSVICAT construct (Stratagene). Clones containing the HAP1 cDNA in the correct orientation were identified using diagnostic restriction digestion.

Stably-transfected rat glioma C6 cells were generated by co-transfection with pOPRSVICAT/HAP1 and p3′SS (which expresses the lac repressor encoded by the *lacl* gene), and selection for resistance to both G418 (750 μg $ml^{-1}$) and hygromycin (20 μg $ml^{-1}$). Cell clones which expressed HAP1 protein following de-repression of the RSV promoter following addition of IPTG (2 mM) were identified by immunoblotting of whole cell extracts.

### Preparation of cell extracts

Following mechanical disruption of adherent monolayers, cells were harvested by centrifugation (1000 rpm, 20°C, 7 min) and washed twice in ice-cold PBS. Protein extracts for immunoblotting were prepared by sonication in 50 mM Tris–HCl pH 8.3, 1 mM EDTA, 3 mM dithiothreitol (DTT) and 1 mM leupeptin; phenylmethylsulphonyl fluoride (PMSF) was added to 1 mM, and cell debris was removed by centrifugation (17 800 ***g***, 4°C, 10 min). Supernatants for AP endonuclease assay were prepared in 50 mM Hepes-KOH, pH 7.5, 150 mM potassium chloride, 10% glycerol and 1 mM DTT containing protease inhibitors (1 μg $ml^{-1}$ aprotinin, 1 μg $ml^{-1}$ pepstatin A, 1 μg $ml^{-1}$ leupeptin A, 100 μg $ml^{-1}$ PMSF) by the same method. Protein concentration was estimated by BioRad protein assay (BioRad), and DNA concentration by Hoechst 33258 fluorimetry using a Hoefer TKO-100 Mini-fluorimeter with excitation and detection wavelengths of 365 nm and 458 nm respectively. Extracts were stored at –80°C.

### Immunoblotting analysis

Cell extracts were prepared as above and aliquots were resolved by sodium dodecyl sulphate polyacrylamide gel electrophoresis (SDS-PAGE) and electroblotted to Hybond C extra membranes (Amersham). The blots were probed with a rabbit anti-HAP1 antiserum (Herring et al, 1998), and then incubated with goat:anti-rabbit IgG conjugated with horseradish peroxidase (Dako). Binding of secondary antibody was detected by enhanced chemiluminescence (Amersham) and exposure to X-ray film (RX, Fuji). Band density was assessed using a UVP Imagestore 5000 system (Ultraviolet Products Ltd, UK), and HAP1 protein was quantitated by comparison with pure rHAP1 protein standards (0–8 ng). HAP1 levels were expressed relative to cell extract DNA content.

© 1999 Cancer Research Campaign

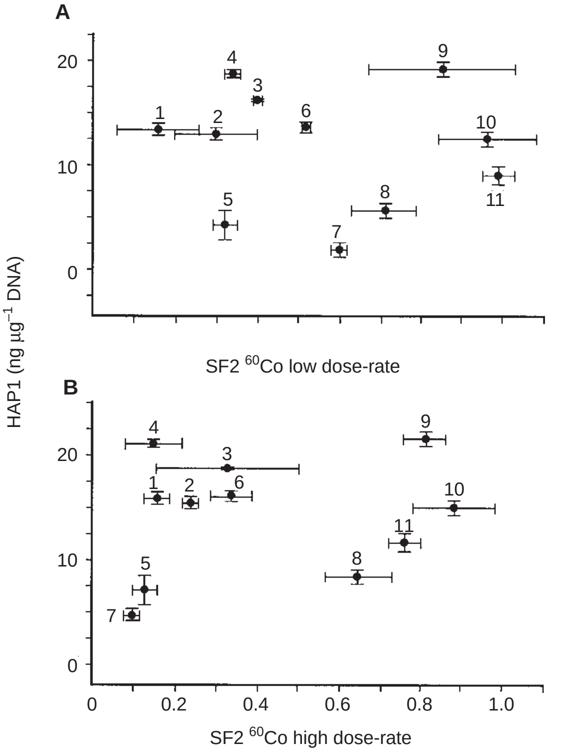

**Figure 1** Comparison between HAP1 protein levels and surviving fraction at 2 Gy (SF2) using high and low dose-rate γ-irradiation. HAP1 expression was determined by western blot analysis and SF2 values were calculated from clonogenic survival curves fitted using the DRFIT program (Roberts, 1990). Errors are ± s.e.m., $n$ = 2–4. No correlation was seen between HAP1 expression and SF2 at high ($r$ = 0.18, $P$ = 0.54) or low dose-rate ($r$ = −0.09, $P$ = 0.78). Cell lines are as follows: 1, A2780; 2, vag12; 3, 350s; 4, nb1-g; 5, ME180; 6, clone F; 7, 351s; 8, WiDr; 9, MS751; 10, HOC8; 11, Du145

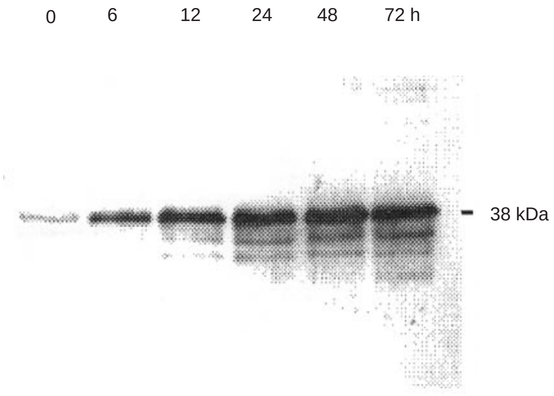

**Figure 2** Immunoblot analysis of HAP1 expression in C6 glioma cells stably transfected with the inducible expression-construct pOPRSVICATHAP1. Expression was induced by treatment of cells with IPTG (0.25 mM) for a period of 0–72 h before harvesting. Cell extracts containing 30 µg protein were analysed by immunoblotting and immunostaining using an anti-human HAP1 specific rabbit serum as described in Materials and Methods. Induction of the 38-kDa HAP1 protein was evident within 6 h, with high levels at 24–72 h after IPTG treatment

### AP endonuclease assay

AP endonuclease activity was determined using an oligonucleotide based assay, essentially as described previously (Winters et al, 1994). A 30-bp synthetic oligonucleotide (5′-TCG GTA CCC GGG GAU CCT CTA GAG TCG ACC-3′), containing a single uracil residue, was 5′-$^{32}$P end-labelled using T4-polynucleotide kinase (PNK) and [γ-$^{32}$P]-ATP. Labelling efficiency was calculated by a DE81 filter binding assay, and unincorporated isotope was removed by Sephadex G-25 spun column chromatography. To create an AP site, unlabelled oligonucleotide (100 pmol) was first mixed with radiolabelled product (500–1000 cps) and then treated with 1 U uracil DNA glycosylase (Boehringer Mannheim) in a buffer containing 60 mM Tris–HCl, 1 mM EDTA, 1 mM DTT, 0.1 mg ml$^{-1}$ BSA, pH 8.0, for 15 min at 37°C. The reaction was terminated by heating at 80°C for 10 min, followed by ethanol precipitation. Exposure of the oligonucleotide to 10% piperidine (90°C, 30 min), PAGE and phosphorimage analysis (Molecular Dynamics, model No. 425) confirmed the AP site status. The HAP1 substrate was generated by annealing of the AP-oligonucleotide to an equimolar quantity of a complementary 30 mer (5′-GGT CGA CTC TAG AGG TTC CCC GGG TAC CGA-3′).

AP endonuclease activity was determined by incubation of cell extract (equivalent to 0–30 ng DNA) with 5 pmol oligonucleotide substrate in a total volume of 25 µl of 50 mM Tris–HCl, pH 8.0, 10 mM sodium chloride, 0.2 mM EDTA, 5 mM magnesium chloride and 50 µg heat inactivated BSA for 5 min at 37°C. The reaction was stopped by the addition of EDTA and proteinase K to 25 mM and 50 µg ml$^{-1}$, respectively, and heating at 42°C for 5 min. Following ethanol precipitation, the oligonucleotide cleavage product was resolved by denaturing PAGE, and quantitated by phosphorimage analysis.

### Clonogenic survival assay

In studies of the induction of wild-type or mutant HAP1 in C6 cells, C6HAP, C6HAP-C65 and C6HAP-D283 cells were treated with 2.5 mM IPTG 24 h prior to γ-irradiation. For low dose-rate irradiation studies, cells growing as monolayers were exposed to 0–8 Gy using a $^{60}$Co source (1.12 cGy per min). Cells were then trypsinized and seeded in triplicate on 6-cm plates at a dilution estimated to give 100 colonies per plate for each dosage point. For high dose-rate irradiation studies, cells were harvested prior to irradiation (0–10 Gy) in suspension using a $^{137}$Cs source (312 cGy per min). After culture for 8–10 days, colonies were fixed in 10% formaldehyde and stained with 0.25% crystal violet before counting. For MMS survival studies, cells were plated 6 h prior to the addition of MMS (0–1.4 mM) and after 1 h exposure the culture medium was replaced.

For human cell lines Du145, WiDr, A2780, HOC8, Ms751, ME180, NB1-g, NB1-clone F, 350s, 351s and vag12, the surviving fraction at 2 Gy (SF2) was determined as a parameter for comparison of cellular radiosensitivity. Survival following low dose-rate ($^{60}$Co 1.12 cGy per min) or high dose-rate (108 cGy per min) irradiation was determined as above for C6 cells. Cells were cultured for 10–20 days before staining and counting.

### RESULTS

### HAP1 expression and radiosensitivity in human cell lines

There was no significant correlation between HAP1 expression, as determined by quantitative immunoblotting, and radiosensitivity

 © 1999 Cancer Research Campaign

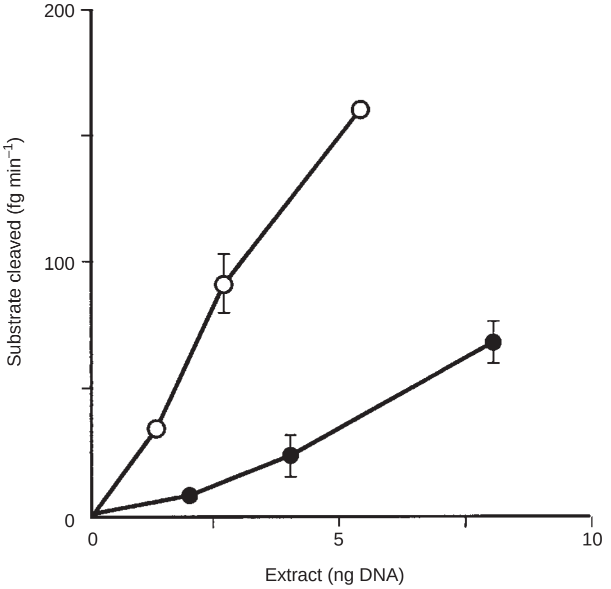

**Figure 3** AP endonuclease activity of C6 cells transfected with the pOPRSVICATHAP1 construct (C6HAP1 cells), demonstrating inducible HAP1 expression. C6HAP1 cells were cultured in the absence (●) or presence (○) of IPTG (0.25 mM, 24 h) and the ability of cell extracts to cleave a $^{32}$P-labelled AP-site containing substrate was determined and expressed relative to cell extract DNA content. Values are the mean of two determinations ± s.d.

characterized by SF2 at either high ($r$ = 0.18, $P$ = 0.54) or low ($r$ = 0.09, $P$ = 0.78) dose-rates (Figure 1). Low dose-rate sparing, calculated by subtraction of the SF2 at high dose-rate from that at low dose-rate for each cell line, also did not correlate well with HAP1 expression (data not shown).

### Effect of HAP1 and mutant HAP1 expression on C6 glioma survival

For C6 cells transfected with the HAP1 expression plasmid, induction of expression of the 38-kDa protein, as assessed by immunoblotting analysis, was evident ~6 h after treatment with IPTG (2.5 mM) and high levels were observed at 24–72 h (Figure 2). After removal of IPTG, HAP1 levels remained elevated for 48 h and could still be detected at 60 h, but decreased to near background levels after 80 h (data not shown). A period of 24 h pretreatment with IPTG was used in subsequent survival studies. In vitro assay of endogenous AP endonuclease activity in extracts of non-induced control C6HAP cells indicated levels that were 74 ± 11% fmoles $h^{-1}$ $\mu g^{-1}$ DNA (s.d., two separate experiments) of those in extracts of 351s cells which had the lowest HAP1 expression among the human cell lines. Following 24 h induction by IPTG, C6HAP AP endonuclease activity was 7.1 ± 1.7-fold (s.e.m., three separate experiments) higher than in non-induced control populations, indicating that active human HAP1 was produced in these cells. The results of a typical assay are shown in Figure 3.

In clonogenic survival assays, induction of HAP1 did not result in increased resistance to γ-irradiation at either high or low dose-rates, or to MMS (Figure 4). C65A and D283A mutant versions of HAP1 have previously been characterized as lacking in redox and DNA repair functions respectively (Walker et al, 1993; Barzilay et al, 1995). Induction of expression of mutant HAP1 proteins on

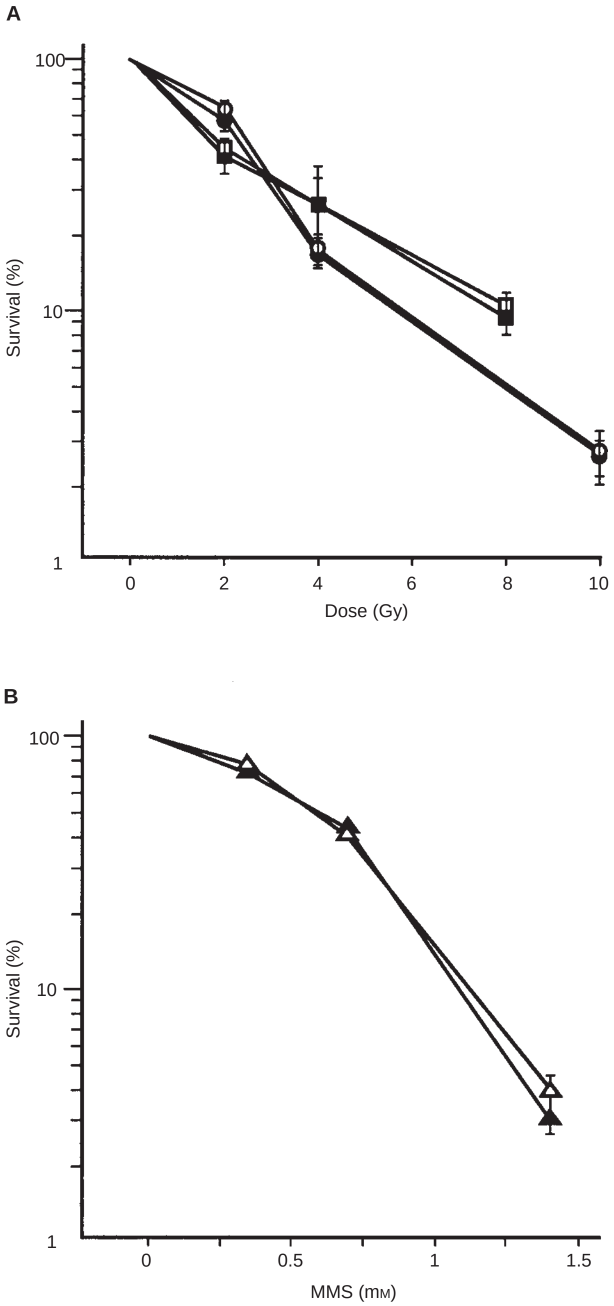

**Figure 4** Survival of C6HAP1 cells following (**A**) high (○, ●), or low (□, ■) dose-rate γ-irradiation and (**B**) treatment with MMS (△, ▲) for IPTG (0.25 mM, 24 h)-induced HAP1 expressing cells (open symbols) relative to non-induced control populations (closed symbols). Points are the mean of at least two independent experiments ± s.d.

treatment of C6HAP:C65A and C6HAP:D283A cells with IPTG (2.5 mM, 24 h) was confirmed by immunoblotting (data not shown), but these cells displayed no altered sensitivity to γ-irradiation at low dose-rates or to MMS (Table 1).

### DISCUSSION

In the present study, we have related the amount of the major AP endonuclease (HAP1) to radiosensitivity in a series of established

*© 1999 Cancer Research Campaign*

**Table 1** Radiation and MMS sensitivity of mutant HAP1 expressing cell lines

| Strain | | MMS SF | | γ-irradiation[a] SF |
|---|---|---|---|---|
| | | 0.7 mM | 1.4 mM | 2 Gy |
| C6HAP:C65A | –IPTG | 0.109 ± 0.015 | 0.013 ± 0.001 | 0.604 ± 0.041 |
| | +IPTG | 0.105 ± 0.008 | 0.019 ± 0.007 | 0.554 ± 0.024 |
| C6HAP:D283A | –IPTG | 0.094 ± 0.026 | 0.004 ± 0.001 | 0.513 ± 0.041 |
| | +IPTG | 0.071 ± 0.013 | 0.003 ± 0.002 | 0.487 ± 0.069 |

[a]Low dose-rate exposure (1.12 cGy per min). Values are the mean for three treated cell populations within a single experiment ± s.d.

human tumour cell lines. HAP1 was chosen because it is the major endonuclease acting on one of the most important consequences of base damage to DNA, i.e. AP sites, following exposure to ionizing radiation: the protein also displays a redox function that promotes the binding of transcription factors to DNA, but the potential role of this in radiosensitivity is not known.

We found no correlation between HAP1 levels and SF2 at either low or high dose-rate among the range of human cell lines examined. This indicates that the levels of HAP1 are not predictive of radiosensitivity among this set of cell lines of various tissue origins, and therefore that, if HAP1 does contribute to cellular protection against γ-irradiation, its role must be relatively minor compared to that of other repair systems.

In order to further address this point, we overexpressed HAP1 in a rat cell line that was relatively deficient in endogenous AP endonuclease activity, but this had no measurable effect on radiation or MMS sensitivity. This is in contrast to an earlier report of a decrease in radiation sensitivity in two radiosensitive murine cell lines following transfection with a HAP1-encoding plasmid (Chen et al, 1992). We therefore also examined the effects on sensitivity to radiation or MMS of overexpression in the rat cell line of two versions of HAP1 that contained inactivating mutations in either the repair or the redox domains (Walker et al, 1993; Barzilay et al, 1995*a*), but neither had any significant effect on radiation or MMS survival. Earlier work had indicated that the expression of *E. coli* endonuclease III in Chinese hamster lung fibroblasts had no effect on sensitivity to ionizing radiation, but it increased sensitivity to the radiomimetic agent bleomycin sulphate, possibly as a consequence of generating more DNA double-strand breaks by cleavage at clustered AP sites (Harrison et al, 1992). On the other hand, expression of endonuclease III in yeast has been shown recently to increase radiation resistance (Skorvaga et al, unpublished data). This was not the case with HAP1 in the present report and since expression of the mutant HAP1 proteins had no detectable effect on low-dose-rate radiosensitivity, it appears that they have no titrational or dominant negative effects on endogenous AP endonuclease activity.

Based on our observations, it is likely that levels of HAP1 do not, in general, correlate with the radiosensitivity of a variety of cell lines. Thus, apart from the enzymes recognizing base modifications and removing them by glycosylase action, the levels of DNA polymerase and ligase might also be rate-limiting in the repair of that radiation damage which has AP sites as intermediates. In addition, any of the many other factors that have been implicated in defining radiosensitivity such as expression of or mutation in ATM, DNA-PK, p53 and other gene products (Yarnold, 1997) may individually or in combination define survival following radiation. Whilst HAP1 may represent one among many factors that are important in toxicity, its effects may only become evident in specific circumstances and in a limited number of cell types. The levels of HAP1 have been reported to be 6–20 times higher in established cell lines than in primary cells (Chen et al, 1991; LaBelle and Linn, 1984), and HAP1 levels may therefore be more critical in the latter. This would be consistent with the significant correlation found between the levels of HAP1 expression detected in fixed tumour biopsies and the radiosensitivity of cells primary-cultured from them (Herring et al, 1998).

### ACKNOWLEDGEMENTS

Work at the PICR was supported by the Cancer Research Campaign and the United Kingdom Co-ordinating Committee on Cancer Research and at Oxford by the Imperial Cancer Research Fund.

### REFERENCES

Barzilay G and Hickson ID (1995) Structure and function of apurinic/apyrimidinic endonucleases. *Bioessays* **17**: 713–719

Barzilay G, Mol CD, Robson CN, Walker LJ, Cunningham RP, Tainer JA and Hickson ID (1995*a*) Identification of critical active-site residues in the multifunctional human DNA repair enzyme HAP1. *Nature Struct Biol* **2**: 561–567

Barzilay G, Walker LJ, Robson CN and Hickson ID (1995*b*) Site-directed mutagenesis of the human DNA repair enzyme HAP1: identification of residues important for AP endonuclease and RNAase H activity. *Nucl Acids Res* **23**: 1544–1550

Barzilay G, Walker LJ, Rothwell DG and Hickson ID (1996) Role of HAP1 protein in repair of oxidative DNA damage and regulation of transcription factors. *Br J Cancer* **74**: S145–S150

Chen DS and Olkowski ZL (1994) Biological responses of human apurinic endonuclease to radiation-induced DNA damage. In *DNA Damage Effects in DNA Structure and Protein Recognition*, *Ann NY Acad Sci* **726** Wallace SS, van Houten B and Kow YW (eds), pp. 306–308.

Chen DS, Herman T and Demple B (1991) Two distinct human DNA diesterases that hydrolyse 3′-blocking deoxyribose fragments from oxidised DNA. *Nucleic Acids Res* **19**: 5907–5914

Chen DS, Herman T and Demple B (1992) Molecular basis for the radiosensitivity of two mouse mutant cell lines. Abstracts of the 40th Annual Meeting of the Radiation Research Society (USA): pp. 21–15

Demple B and Harrison L (1994) Repair of oxidative damage to DNA: enzymology and biology. *Ann Rev Biochem* **63**: 915–948

Demple B, Johnson A and Fung D (1986) Exonuclease III and endonuclease IV remove 3′ blocks from DNA synthesis primers in $H_2O_2$-damaged *Escherichia coli*. *Proc Natl Acad Sci USA* **83**, 7731–7735

Demple B, Herman T and Chen DS (1991) Cloning and expression of APE, the cDNA encoding the major human apurinic endonuclease: definition of a family of DNA repair enzymes. *Proc Natl Acad Sci USA* **88**: 11450–11454

Doetsch PW and Cunningham RP (1990) The enzymology of apurinic/apyrimidinic endonucleases. *Mutat Res* **236**: 173–201

Harrison L, Skorvaga M, Cunningham RP, Hendry JH and Margison GP (1992) Transfection of the *E. coli nth* gene into radiosensitive Chinese hamster cells: effects on sensitivity to radiation, hydrogen peroxide and bleomycin sulphate. *Radiat Res* **132**: 30–39

Henner WD, Grunberg SM and Haseltine WA (1983) Enzyme action at 3′ termini of ionizing radiation-induced DNA strand breaks. *J Biol Chem* **258**: 15198–15205

Herring CJ, West CML, Wilks DP, Davidson SE, Hunter RD, Berry P, Forster G, McKinnon J, Rafferty JA, Elder RH, Hendry JH and Margison GP (1998) Levels of the DNA repair enzyme HAP1(APE1,Apex, Ref-1) are associated with the intrinsic radiosensitivity of cervix cancers. *Br J Cancer* **78**: 1128–1133

Jayaraman L, Murthy KG, Zhu C, Curran T, Xanthoudakis S and Prives C (1997) Identification of redox/repair protein Ref-1 as a potent activator of p53. *Genes Dev* **11**: 558–570

LaBelle M and Linn S (1984) DNA repair in cultured mouse cells of increasing population doubling level. *Mutat Res* **132**: 51–61

 © 1999 Cancer Research Campaign

Lindahl T (1990) Repair of intrinsic DNA lesions. *Mutat Res* **238**: 305–311
Lindahl T and Andersson A (1972) Rate of chain breakage at apurinic sites in double-stranded deoxyribonucleic acid. *Biochemistry* **11**: 3618–3623
Loeb LA and Preston BD (1986) Mutagenesis by apurinic/apyrimidinic sites. *Ann Rev Genet* **20**: 201–230
Ono Y, Furuta T, Ohmoto T, Akiyama K and Seki S (1994) Stable expression in rat glioma cells of sense and antisense nucleic acids to a human multifunctional DNA repair enzyme, APEX nuclease. *Mutat Res DNA Repair* **315**: 55–63
Ono Y, Matsumoto K, Furuta T, Ohmoto T, Akiyama K and Seki S (1995) Relationship between expression of a major apurinic/apyrimidinic endonuclease (APEX nuclease) and susceptibility to genotoxic agents in human glioma cell lines. *J Neuro-Oncol* **25**: 183–192
Roberts SA (1990) DRFIT: a program for fitting radiation survival models. *Int J Radiat Biol* **57**: 1243–1246
Robson CN and Hickson ID (1991) Isolation of cDNA clones encoding a human apurinic/apyrimidinic endonuclease that corrects DNA repair and mutagenesis defects in *E. coli* xth (exonuclease III) mutants. *Nuceicl Acids Res* **19**: 5519–5523
Rothwell DG and Hickson ID (1996) Asparagine 212 is essential for abasic site recognition by the human DNA repair endonuclease HAP1. *Nucleic Acids Res* **24**: 4217–4221
Rothwell DG, Barzilay G, Gorman M, Morera S, Freemont P and Hickson ID (1997) The structure and functions of the HAP1/Ref-1 protein. *Oncol Res* **9**: 275–280
Seki S, Ikdea S, Watanabe S, Hatsushika M, Tsutsui K, Akiyama K and Zhang B (1991) A mouse DNA repair enzyme (APEX nuclease), having exonuclease and apurinic/apyrimidinic endonuclease activities: purification and characterisation. *Biochim Biophys Acta* **1079**: 57–64
Teoule R (1987) Radiation-induced DNA damage and its repair. *Int J Radiat Biol* **51**: 573–589
Walker LJ, Robson CN, Black E, Gillespie D and Hickson ID (1993) Identification of residues in the human DNA repair enzyme HAP1 (Ref-1) that are essential for redox regulation of Jun DNA binding. *Mol Cell Biol* **13**: 5370–5376
Walker LJ, Craig RB, Harris AL and Hickson ID (1994) A role for human DNA repair enzyme HAP1 in cellular protection against DNA damaging agents and hypoxic stress. *Nucleic Acids Res* **22**: 4884–4889
Ward JF, Jones GDD, Milligan JR (1994) Biological consequences of non-homogeneous energy deposition by ionising radiation. *Radiat Prot Dosim* **52**: 271–276
Winters TA, Henner WD, Russell SP, McCullough A and Jorgensen TJ (1994) Removal of 3′-phosphoglycolate from DNA strand-break damage in an oligonucleotide substrate by recombinant human apurinic/apyrimidinic endonuclease 1. *Nucleic Acids Res* **22**: 1866–1873
Xanthoudakis S and Curran T (1992) Identification and characterisation of Ref-1, a nuclear protein that facilitates AP-1 DNA-binding activity. *EMBO J* **11**: 653–665
Xanthoudakis S, Miao G, Wang F, E.Pan, Y-C and Curran T (1992) Redox activation of Fos-Jun DNA binding activity is mediated by a DNA repair enzyme. *EMBO J* **11**: 3323–3335
Xanthoudakis S, Miao GG and Curran T (1994) The redox and DNA-repair activities of Ref-1 are encoded by nonoverlapping domains. *Proc Natl Acad Sci USA* **91**: 23–27
Yarnold J (1997) Molecular aspects of cellular responses to radiotherapy. *Radiother Oncol* **44**: 1–7

*© 1999 Cancer Research Campaign*
